# Accuracy of a smartphone-based 3D imaging tool for height measurement and stunting detection among children 24–59 months of age in Nepal: a validation study

**DOI:** 10.1016/j.lansea.2026.100774

**Published:** 2026-04-24

**Authors:** Ritika Mukherjee, Shivangi Kaushik, Hamza Salah, Ankit Kumar Gupta, Divita Sharma, Miriam Kahraman, Anupama Vadyadagaya, Nasrullah Ali, Mahesh Rijal, Sampurna Rai, Archisman Mohapatra

**Affiliations:** a Generating Research Insights for Development Council (GRID Council), H 54, Sector 63, Noida, Uttar Pradesh, 201309, India; b Welthungerhilfe, Deutsche Welthungerhilfe e.V. Friedrich-Ebert-Straße 1, 53173, Bonn, Germany; c Child Growth Monitor, Welthungerhilfe, House No- 144, Shivam Footwear, Banda Road, Atarra, Banda, Uttar Pradesh, 210201, India; d Deutsche Welthungerhilfe e.V., Deutsche Welthungerhilfe e.V. Friedrich-Ebert-Straße 1, 53173, Bonn, Germany; e Child Growth Monitor, WHH, #12-513D1, Bantwal, Dakshina Kannada, Karnataka, 574219, India; f Welthungerhilfe Nepal, 341, Kalika Mandir Marga, Sanepa, Lalitpur - 02, Nepal; g NTAG, Ukti Marg, Maitighar Height Kathmandu, Bagmati, 44600, Nepal

**Keywords:** Artificial intelligence, Digital anthropometry, Child nutrition, Malnutrition, Health technology, Growth monitoring, South-East Asia

## Abstract

**Background:**

Detection of stunting in young children using traditional anthropometry has been a challenge at the community level especially in resource-constrained settings. We evaluated the performance of Child Growth Monitor (CGM), a smartphone-based 3D depth-imaging application, for screening of stunting, and height measurement among children (24–59 months of age) in Nepal.

**Methods:**

At four health-posts in Rautahat district, Nepal, in October 2024, we conducted cross-sectional validation study comparing CGM's stunting detection against gold standard (manual measurement) and prospective reliability assessment with repeat measurements, four days apart. Eight trained enumerators collected data using calibrated height boards and CGM app. Validity was assessed through sensitivity, specificity and accuracy, reliability through intraclass correlation coefficients (ICC) and Technical Error of Measurement (TEM). Pitman's test evaluated variance. Bias was assessed using Bland Altman statistics.

**Findings:**

310 children were recruited, with analyses based on paired subsets (n = 261). Manual measurements identified 36% as stunted, while CGM detected 31·8%. CGM demonstrated 84·0% sensitivity, 98·2% specificity, 96·3% positive and 91·6% negative predictive values, and 93·1% accuracy. For height measurement, the ICC for intra-rater and inter-rater reliability were 0·990 (n = 76) and 0·992 (n = 75) respectively, with intra- and inter-rater TEM of 0·5 cm. Inter-method TEM was 0·7 cm. Pitman's test was significant; Bland–Altman showed mean bias of 0·3 cm.

**Interpretation:**

CGM demonstrated accuracy and reliability for field-based stunting detection among children belonging to 24–59 months of age in Nepal. Future research on its use in younger children (6–24 months) and diverse settings will enable its integration into routine growth monitoring.

**Funding:**

This study was funded by the German Federal Foreign Office as part of the overall funding for the development of Child Growth Monitor app.

**Research in context**

**Evidence before this study**

Malnutrition, particularly stunting among children less than five years of age, remains a major global health challenge, with highest burden in Southeast Asia. Accurate assessment of child growth is essential for early identification and management of child malnutrition. While traditional anthropometry using WHO growth standards is widely practiced, it remains prone to manual error, requires trained personnel, and is often not feasible in remote or resource-limited settings. We searched PubMed up to January 2025 using combinations of the keywords “child growth monitoring,” “stunting detection,” “mobile application,” “artificial intelligence,” and “low-middle income countries” to identify studies evaluating digital tools for child growth assessment. The available evidence suggests that emerging technologies like mobile applications offer promising solutions by enabling portable, contactless, and standardised growth assessments. A few studies have explored mobile or AI-enabled technologies to support growth monitoring, but evidence on their diagnostic performance and validity remains limited.

**Added value of this study**

This study evaluates the diagnostic performance of the Child Growth Monitor (CGM) application (app), a digital tool developed by Welthungerhilfe e.V. (WHH) to detect stunting among children aged 24–59 months. The CGM app utilises smartphone-based artificial intelligence and deep learning algorithms, leveraging a Time-of-Flight (ToF) sensor to predict height from depth maps and RGB images captured in multiple poses. The study demonstrates the app's diagnostic accuracy with strong sensitivity and specificity in identifying stunting. It also provides key reliability metrics, such as intra-class correlation and technical error of measurement (TEM), indicating consistent performance across trained enumerators. The study highlights the feasibility of deploying smartphone-based, image-driven growth assessment tools at the community level, especially in contexts where health workers face infrastructure and capacity limitations.

**Implications of all the available evidence**

The adoption of digital solutions like the CGM app has the potential to transform child malnutrition screening by addressing limitations in manual anthropometry. If validated for accuracy and reliability, these technologies can enhance early stunting detection, enable timely interventions, and improve health outcomes. The integration of AI-powered digital tools into routine health monitoring programs can facilitate data-driven decision-making, reduce measurement inconsistencies, and optimise resource allocation for public health nutrition initiatives. Further research is warranted to assess cost-effectiveness, scalability, and long-term impact on health outcomes.

## Introduction

Malnutrition remains a significant global health challenge, particularly for children under five years of age.^1^^,^^2^ Among its various manifestations, stunting is considered as a key indicator of chronic undernutrition. It has lifelong consequences affecting physical growth, cognitive development, and overall health of children.^3^, ^4^, ^5^ An estimated 22·3% (∼148·1 millions) of children under five years were stunted worldwide, with prevalence disproportionately higher in South Asia (30·7%) and Nepal (31·5%).^6^^,^^7^ Beyond health implications, stunting undermines economic growth by limiting education attainment, workforce productivity, and lifetime earnings.^8^, ^9^, ^10^, ^11^ In some countries, its economic burden accounts for annual losses of 0·01% to 1·2% of national Gross Domestic Product (GDP), highlighting the need for effective intervention by policymakers.^8^, ^9^, ^10^ Accurate height measurement is crucial for identifying stunting, and children between 24 and 59 months of age are particularly important for assessment.^12^, ^13^, ^14^, ^15^ This age group represents a critical period for assessing the cumulative impact of nutritional and health interventions, which can affect long-term growth and development.^16^^,^^17^ Children in this age group tend to be more cooperative during anthropometric measurements, improving data quality in public health nutrition assessments.^18^ However, in resource-constrained field settings, traditional anthropometric measurements face challenges like human error, workforce shortages and lack of standardisation, limiting their utility for large-scale malnutrition surveillance and intervention planning.^11^^,^^19^, ^20^, ^21^, ^22^, ^23^, ^24^

Advances in digital health technology, especially mobile applications, offer promising solutions for overcoming these challenges. Portable, contactless, and standardised tools have demonstrated modest potential in improving operational efficiency and accuracy in low-resource settings.^25^^,^^26^ However, their reliability and validity in field settings remain inadequately evaluated against gold standard anthropometry, making their real-world applicability uncertain.^27^^,^^28^

The Child Growth Monitor application (CGM app) developed by Welthungerhilfe e.V. (WHH) uses smartphone-based artificial intelligence (AI) technology and Time-of-Flight (ToF) sensor to capture depth maps and red-green-blue (RGB) images in four poses.^29^^,^^30^ Details of ToF is given in Supplementary File 2. AI algorithm reconstructs 3D body model and deep learning algorithms then predict height, generating standardised estimates that are automatically plotted on World Health Organisation (WHO) growth charts, thus providing digitised malnutrition detection. While this tool offers a rapid and scalable approach for detecting malnutrition, its screening performance warrants rigorous evaluation. The 3D based scanning may reduce observer-dependent error and enable non-contact, full-body capture, whereas manual measurements rely on enumerator skill and consistency. However, accurate scanning also depends on proper device handling and adherence to standardised protocols, similar to conventional anthropometry. Emerging evidence suggests that such 3D imaging tools demonstrate acceptable validity and reliability for anthropometric assessment, supporting their potential for use in population-level screening.^31^^,^^32^ Manual anthropometry in children often suffers from technical errors and variability among measurers despite training. Applications (app) or automated systems can reduce such errors, but they require validation. Training manual assessors demands significant time and resources compared to app deployment in resource-limited settings.^22^^,^^33^^,^^34^

Therefore, this study aimed to evaluate the performance of CGM app for screening stunting, and measurement of height, among children between 24 and 59 months of age in resource-constrained field settings. The findings will help in providing evidence-based insights into its potential integration into routine malnutrition surveillance and public health programs in similar settings.

## Methods

A validation study design with two components was used in Rautahat district in Nepal, a region with high burden of malnutrition.^35^ First, a cross-sectional validation was carried out to assess criterion validity of the tool by comparing its performance for detecting stunting with those obtained using the established gold standard (manual method) at baseline. Second, a prospective reliability assessment was undertaken using repeat measurements. The district was selected purposively considering WHH's long-standing nutrition projects and strong community rapport, facilitating community-based recruitment. Using purposive sampling, four Health Posts (HP) i.e., primary-level health facilities were identified from eight villages across the district. Selection criteria considered estimated numbers of children between 24 and 59 months of age in the villages and area-specific malnutrition prevalence rates.

Children between 24 and 59 months of age, residing in the selected villages and registered within four HP catchment areas were eligible for inclusion. The Village Health Volunteers (VHV) of each HP conducted household visits and invited eligible children for participation based on their existing health records. Eligibility of the children was confirmed through their birth certificates and/or immunisation card. Efforts were made to include both boys and girls in similar numbers and across visible nutritional profiles (apparently stunted, wasted, obese, normal nutritional status). Children with acute illnesses or those unable to cooperate with height measurements were excluded.

Assuming 90% sensitivity (Sn) of CGM app for detecting children with stunting, relative precision of 10%, 95% confidence level (CI), and 20% data loss, a sample size of 52 children with stunting was calculated. Assuming the prevalence of stunting to be about 20%, a survey of about 260 children was required. To round off, a sample size of 300 children between 24 and 59 months of age were targeted for inclusion in the study. For assessing the reliability of repeated measurements, a 40% sub-sample (n = 120) was re-tested four days after the initial measurement.

Enumerators responsible for measuring height (manual and CGM app) of children were recruited based on the predefined criteria i.e., minimum secondary education, prior experience of conducting surveys and anthropometry, and fluency in Nepali and local dialects. All selected candidates underwent a 3-day Standardised Monitoring and Assessment of Relief and Transitions (SMART) training, led by one of the authors (SK) (SMART Survey Manager certified ≥10 years of experience), covering standard height measurement technique, use of CGM app and mobile device handling. Hands-on practice session was followed by evaluation. Details of training have been provided in Supplementary File 3. The final selection was based on the performance (acceptable intra-enumerator technical error of measurement (TEM) < 0·6 cm) of the enumerators.^21^^,^^25^^,^^36^, ^37^, ^38^, ^39^, ^40^ Four male enumerators (nutritionists with ≥5 years' experience) and four assistants (two male, two female with ≥7 years’ experience) between 29 and 35 years of age were selected.

Data collection spanned over nine days (October 20th–28th, 2024) across the four health posts (HPs: I, II, III, IV). Each HP was assigned one trained enumerator and one assistant. During the first five days, all four HPs were operational. Height measurements were taken for at least 15 children per HP per day, such that at least 300 children were measured by the end of Day 5. Each child was first scanned with the CGM app, then measured manually using a UNICEF validated height board (1–2 cm thick, light wood) by the assigned enumerator. Each HP measured at least 15 children per day, targeting repeat measurement for 120 children over the next four days (Supplementary File 4: Table S4.1). Each child underwent repeat measurements by their original enumerator (first by CGM app then manually), followed by the same process with a second enumerator (Supplementary File 5: Figure S5.1). All the enumerators were blinded to the CGM app results and to each other's measurements. At the HP, each child was first verified using the VHV's child list. Parents/caregivers were then informed about the process and asked to prepare the child for assessment (stands upright with feet together, minimal clothing, and hair was tied properly, no loose or messy hair or pony tail) and they were requested to stay with the child to ensure cooperation and comfort.^41^ Efforts were made to maintain consistent and sufficient lighting conditions with clear background (no light absorbing objects or surfaces) across sites by utilising naturally lit indoor spaces and avoiding direct sunlight exposure during scanning, as excessive or variable lighting may affect depth image quality. Enumerators were trained to position children and the device appropriately to minimise shadows and ensure optimal capture conditions. The application was also available in Nepalese along with English for the ease of data collection.

The CGM app was designed with robust security measures to ensure data integrity and confidentiality. Data was encrypted locally on devices before transmission and securely uploaded to a central database via Hypertext Transfer Protocol Secure (HTTPS). Offline functionality allowed data collection in areas with limited internet, with a remote wipe feature enabling data deletion if devices were lost. Pictures were automatically deleted from phones after being uploaded to the servers. Data security included encryption at rest for databases and file storage, secure HTTPS/Secure Sockets Layer (SSL) transmission, and user authentication via Azure B2C. App access was restricted to verified users through manual verification. CGM app downloads were limited to authorised users via play store setting. Regular encrypted backups safeguarded against data loss. This multi-layered security approach ensured data protection during collection, storage, and transmission.^42^

### The approach and metrics for data analysis were guided by WHO Multicentre

Growth Reference Study (MGRS), SMART Methodology and UNICEF Target Product Profile Tools for Anthropometric Measurements. The anonymised dataset shared with the research team (GRID) included unique child identifiers, gender, age, date and time of measurement, CGM app's device number, values for manual and CGM app's height measurements, along with height-for-age z-scores (HAZ) and CGM based classification of stunting. The quality of data was assessed to ensure the plausibility of HAZ. Implausible values were flagged using WHO macro criteria (HAZ < −6 or >6) and Demographic and Health Surveys (DHS) plausibility checks (height outside 65–120 cm).^43^

### Statistical analysis

Statistical analysis assessed validity, bias, and reliability. Test-retest reliability of manual measurement was done. Validity was evaluated by comparing CGM app and manual measurements (gold standard) using intraclass–correlation coefficient (ICC), Spearman-Brown Split–Half coefficient, and screening performance metrics (sensitivity [Sn], specificity [Sp], positive and negative predictive values [PPV and NPV, respectively] and likelihood ratio [LR+ and LR-] and accuracy). Receiver Operating Characteristic (ROC) curve and Lin's Concordance Correlation analysis evaluated CGM app's area under curve (AUC) for discrimination and precision, respectively, across enumerators.

Reliability was tested through intra-rater (same enumerator, different occasions) and inter-rater (different enumerators, same occasion) testing using scatter plots, ICC, and TEM. Variance was assessed using Pitman test. Cohen's Kappa was used to assess agreement for CGM based stunting detection. Bias was analysed via Bland–Altman, Cumulative Sum (CUSUM) test, and spline regression (due to non-linearity of data). Statistical significance was set at p < 0·05. Analysis was performed using R Studio 4·3·3.

Personally identifiable information (PII) was removed before processing, ensuring anonymisation and responsible data analysis. All manual measurements were received from Nepali Technical Assistance Group (NTAG) and all scan data were received from the WHH team. Daily calibration of height boards (using a 110 cm reference stick) and phone accuracy checks (via a calibration image) ensured measurement reliability. This study follows the Standards for Reporting of Diagnostic Accuracy Studies (STARD) reporting guidelines.^44^

### Ethics statement

Ethical approval for this study was granted by the Nepal Health Research Council (NHRC) (Reference number 673, 24 September 2024). Children's rights and privacy were safeguarded through a two-stage informed consent process, ensuring compliance with local and international ethical standards. Prior to data collection, caregivers were provided with detailed information regarding the study objectives, voluntary participation, data handling procedures, image processing, and the right to withdraw or request data deletion at any stage. Written informed consent was obtained for both initial and repeat measurements.

### Role of the funding source

The funders had no role in study design, data collection, analysis, or interpretation, participant recruitment or any aspect pertinent to the study.

## Results

A total of 310 children (51·0% girls and 49·0% boys) participated in the first measurement. Among them, 60 (19·4%) were 24–35 months, 136 (43·8%) were 36–47 months, and 114 (36·8%) were 48–59 months of age. Manual height data was available for 309 (99·7%). Both manual and CGM app's height data were available for 261 (84·2%) children. None of the assessed children were identified with oedema. For 47 children (15·2%), the CGM app was unable to generate data due to poor-quality depth maps caused by faulty devices.

Based on manual height measurement, 198 (64·1%) were classified as not stunted, 91 (29·4%) were moderately stunted, and 20 (6·5%) were severely stunted. In comparison, CGM app classified 179 (68·1%) as not stunted, 72 (27·4%) as moderately stunted and 12 (4·6%) as severely stunted (Supplementary File 4: Table S4.2–S4.4).

**Fig. 1:**
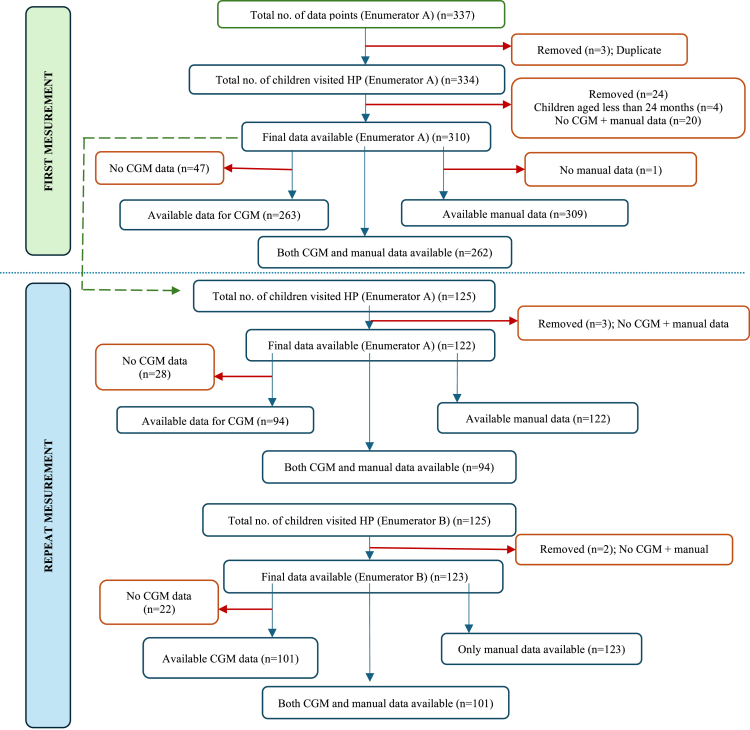
Data collection flowchart.

Only 125 (40·3%) children returned for repeat measurement. Among them repeat measurement data by the same enumerator as that of first day was available for 122 children. Data for repeat measurements by different enumerator from that of first day was available for 123 children. For repeat measurements, paired CGM app and manual data by the same enumerator were available for 94 (77%) children and paired data by different enumerator was available for 101 (82·1%) children (Fig. 1) (Supplementary File 4: Table S4.5). No HAZ scores were flagged as implausible based on WHO criteria (range: −4·3 to 3·8) and DHS thresholds (range: 77·5 to 110·2 cm). One erroneous data point was excluded during data cleaning.

For assessing the screening performance and bias, data was available for 261 children. The ICC for test-retest reliability of manual height measurement using UNICEF validated height board (gold standard) was 0·949 (95% CI: 0·928–0·964, p-value <0·001; n = 122).

The overall screening performance of CGM app's measurement was compared to gold standard. CGM app showed sensitivity of 84% (95% CI: 75·1–90·8) and specificity of 98·2% (95% CI: 94·8–99·6). The PPV of CGM app was 96·3% (95% CI: 89·7–99·2) while the NPV was 91·6% (95% CI: 86·6–95·2) (Tables 1 and 2).

**Table 1:** Contingency table of overall diagnostic performance of CGM app with gold standard in detection of stunting (n = 261).

**CGM app measurement**	**Manual measurement (Gold standard)**			

	**Diagnosis**	**Stunted**	**Not stunted**	**Total**

	Stunted	79	3	82
	Not stunted	15	164	179
	Total	94	167	261

Note: Stunted includes all categories of stunting.

**Table 2:** Diagnostic performance of CGM app with gold standard.

Profile	CGM stunted n (%)	Gold standard stunted n (%)	Sn (%) (95% CI)	Sp (%) (95% CI)	PPV (%) (95% CI)	NPV (%) (95% CI)	LR+	LR-	Accuracy (%) (95% CI)
Overall diagnostic performance									
CGM vs Gold standard (Manual) (n = 261)	83 (31·8)	94 (36·0)	84·0 (75·1–90·8)	98·2 (94·8–99·6)	96·3 (89·7–99·2)	91·6 (86·6–95·2)	46·7	0·16	93·1 (89·3–95·9)
Nutritional status									
Not wasted AND not underweight ± stunted (n = 152)^b^	18 (11·8)	23 (15·1)	73·9 (51·6–89·8)	99·2 (95·8–99·9)	94·4 (72·7–99·9)	95·5 (90·5–98·3)	94·8	0·3	95·4 (90·7–98·1)
Wasted AND/OR underweight ± stunted (n = 109)^c^	64 (58·7)	71 (65·1)	87·3 (77·3–94)	94·7 (82·2–99·4)	96·9 (89·2–99.6)	80·0 (65·4–90·4)	16·6	0·1	89·9 (82·7–94·8)
Wasted AND underweight ± stunted (n = 35)^d^	16 (45·7)	20 (57·1)	75·0 (50·9–91·3)	93·3 (68–99·8)	93·7 (69·8–99·8)	73·7 (48·8–90·8)	11·2	0·3	82·9 (66·3–93·4)
Not stunted + Severely stunted (n = 178)^e^	11 (6·2)	11 (6·2)	100	100	100	100	Inf^a^	0	100
Not stunted + Moderately stunted (n = 246)^f^	67 (27·2)	79 (32·1)	81·0 (70·6–88)	98·2 (94·8–99·6)	95·5 (87·5–99·1)	91·6 (86·6–95·2)	45·0	0·2	92·7 (88·7–95·6)
Gender									
Boys (n = 129)	38 (29·5)	42 (32·6)	85·7 (71·5–94·6)	97·7 (91·9–99·7)	94·7 (82·2–99·4)	93·4 (86·2–97·5)	37·3	0·1	93·8 (88·1–97·3)
Girls (n = 132)	44 (33·3)	52 (39·4)	82·7 (69·7–91·8)	98·7 (93·2–99)	97·7 (87–99·9)	89·8 (81·5–95·2)	66·1	0·2	92·4 (86·5–96·3)
Age (in months)									
24–35 months (n = 41)	10 (24·4)	14 (34·1)	64·3 (35·1–87·2)	96·3 (81–99·9)	90 (55·5–99·7)	83·9 (66·3–94·5)	17·4	0·4	85·4 (70·8–94·4)
36–47 months (n = 123)	36 (29·3)	40 (32·5)	87·5 (73·2–95·8)	98·8 (93·5–99)	97·2 (85·5–99·9)	94·2 (87·1–98·1)	72·9	0·1	95·1 (89·7–98·2)
48–59 months (n = 97)	36 (37·1)	40 (41·2)	87·5 (73·2–95·8)	98·2 (90·6–99)	97·2 (85·5–99·9)	91·8 (81·9–97·3)	50	0·1	93·8 (87–97·7)

a Inf: Infinite.

b Children who were either stunted or not stunted but had normal nutritional status for wasting and underweight.

c Children who were either stunted or not stunted but exhibited one or both conditions of wasting and underweight.

d Children who were either stunted or not stunted and exhibited both wasting and underweight, indicating severe nutritional challenges.

e Children who were either severely stunted or not stunted.

f Children who were either moderately stunted or not stunted.

**Fig. 2:**
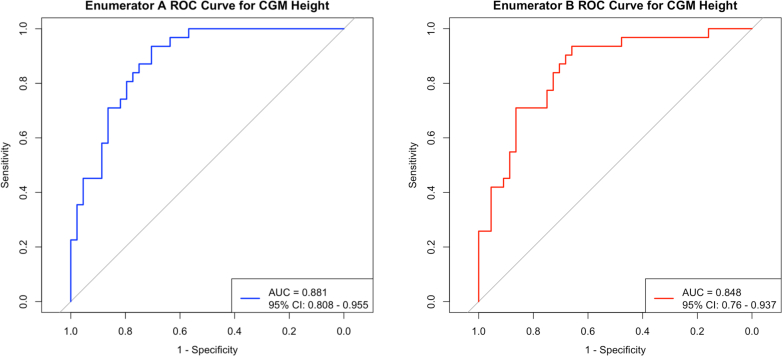
ROC plot of Enumerator A and Enumerator B.

The AUC for discrimination and precision of CGM app measurements between two enumerators (A and B) were evaluated using ROC analysis and Lin's CCC, respectively, for 75 children. The ROC analysis demonstrated area under curve (AUC) of 0·881 (95% CI: 0·808–0·955) (Enumerator A) and 0·848 (Enumerator B) (95% CI: 0·760–0·937) (Fig. 2) (Supplementary File 4: Table S4.6). Lin's CCC of Enumerator A was 0·990 (95% CI: 0·984–0·994) and of Enumerator B was 0·978 (95% CI: 0·965–0·986).

**Fig. 3:**
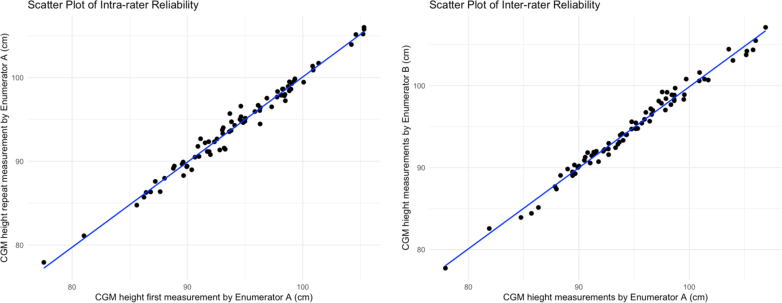
Scatter plots of intra-rater reliability & inter-rater reliability of CGM app for prediction of height.

Data was available for measuring intra- and inter-rater reliability for 76 and 75 children, respectively. For intra-rater reliability, CGM app showed an ICC of 0·990 (95% CI: 0·984–0·994), paired t-test showed no significant results (p = 0·882) and intra-TEM was 0·5 cm (relative TEM of 0·6%). Inter-rater reliability for CGM app showed ICC of 0·992 (95% CI: 0·987–0·995, paired t-test, p = 0·329) with inter-TEM of 0·5 cm (relative TEM of 0·5%) (Table 3) (Fig. 3). The CGM app showed consistently high intra- and inter-rater reliability across gender (Boy, Girl) and age strata (24–35 months, 36–47 months and 48–59 months) (ICCs >0·98 for all groups; p < 0·001) (Supplementary File 4: Table S4.7–S4.8). The inter-method TEM between CGM app and manual height measurements was 0.7 cm (relative TEM: 0·8%; mean: 95·1 cm; r: 0·986). Agreement on stunting detection was calculated using kappa statistics between the two enumerators for CGM app measurements indicating very good agreement. Pitman's test showed significant result (Supplementary File 4: Table S4.9).

**Table 3:** Performance (reliability) of CGM app and manual measurement for prediction of height.

Reliability	Mean height (cm)		Technical error of measurement (TEM) (cm)		Relative TEM (%TEM)		Coefficient of reliability in technical error of measurement (R)		Intraclass correlation coefficient (95% CI)		Paired t test (p-value)	
	CGM	Manual	CGM	Manual	CGM	Manual	CGM	Manual	CGM	Manual	CGM	Manual
Intra rater (n = 76)	94·3	93·9	0·5	0·3	0·6	0·3	0·990	0·997	0·990 (0·984–0·994)^a^	0·997 (0·996–0·998)^a^	0·837	0·609
Inter rater (n = 75)	94·7	94·3	0·5	0·7	0·5	0·7	0·992	0·985	0·992 (0·987–0·995)^a^	0·985 (0·977–0·991)^a^	0·329	0·156

a Statistically significant p < 0·05.

**Fig. 4:**
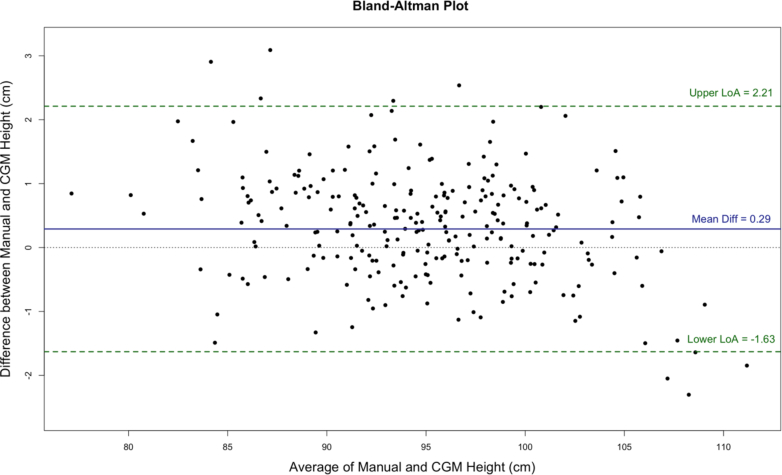
Bland–Altman plots of CGM app height with manual height measurement.

Agreement between CGM app and manual height measurements was assessed on children who were measured by enumerator A on first day (n = 261). The Passing-Bablok regression was applied but CUSUM test revealed a significant deviation from linearity (p < 0·001) (Supplementary File 4: Table S4.9). Spline regression showed significant results (intercept: 77·9 cm, 95% CI: 76·9–78·8; p < 0·001). Bland–Altman analysis showed a mean bias of 0·3 cm (95% CI: 0·2–0·4), with lower limit of agreement of −1·6 cm (95% CI: −1·8 to −1·4) and upper limit of agreement of 2·2 cm (95% CI: 2–2·4) (Fig. 4). The ICC and Spearman-Brown Split–Half coefficient was calculated between CGM and gold standard measurements (Supplementary File 4: Table S4.10).

## Discussion

This study aimed to evaluate the performance of CGM app for height measurement and stunting detection among children between 24 and 59 months of age, using manual measurement as gold standard. After accounting for duplicates and missing data, the study achieved a sample size of 309 children. Completeness of the data was high, with manual measurements available for 99·7% of children and CGM app's data successfully obtained for 84·2% of the children. The findings suggest that the CGM app had excellent reliability and screening accuracy, with promising implications for its use in child growth monitoring.

The CGM app slightly overestimated height measurements, a trend consistent with the findings from other studies evaluating automated anthropometric tools.^25^^,^^27^ Length of the scalp hair could be a potential reason for this.^45^ Despite this, the app demonstrated excellent intra- and inter-rater reliability (ICC >0·990) and high precision with lower relative TEM, aligning with previous research on digital anthropometric tools used in field settings.^40^^,^^46^ The strong agreement in measurements across different enumerators indicates the app's ability to provide consistent and reproducible results, making it a promising tool for large-scale deployment. The CGM app's Sn (84·0%, 95% CI: 75·1–90·8), Sp (98·2%, 95% CI: 94·8–99·6), PPV (96·3%, 95% CI: 89·7–99·2) and NPV (91·6%, 95% CI: 86·6–95·2) confirmed its accuracy and effectiveness for screening children for stunting at the frontline. The app's stable performance across age, gender, and nutritional subgroups may be considered as an improvement over some of the previously tested tools.^39^^,^^40^ The app's overall screening performance supports its integration into public health systems as an efficient and scalable solution for nutrition monitoring and optimised referral.

However, the study also had certain limitations. The CGM app's reliance on specific positioning and orientation, coupled with sensitivity to background conditions, contributed to higher proportions of missed measurements (1st visit: 20·3%, 95% CI: 16·1–25·1; 2nd visit: Enumerator A: 24·8%, 95% CI: 17·5–33·3; Enumerator B: 19·2%, 95% CI: 12·7–27·2) compared to manual methods (1st visit: 6·4%, 95% CI: 4·0–9·6; 2nd visit: Enumerator A: 2·4%, 95% CI: 0·5–6·9; Enumerator B: 1·6%, 95% CI: 0·2–5·7). Similar challenges have been reported with other relatable technologies.^24^^,^^40^^,^^47^ In most of these missed cases, the RGB image quality from CGM was good, which makes the restoration process potentially feasible using RGB-based depth map conversion models in future studies. Children under 24 months were excluded from this study as the CGM app requires the child to stand still for accurate measurement. Since recumbent length is typically used for children under 24 months,^47^^,^^48^ more research is needed for this age group following recent updates to the app. Inclusion of children between 24 and 59 months limits the scalability for early detection of stunting. Measurement-specific barriers included difficulty in ensuring children maintained correct posture, and limited private spaces. Measurement order was not randomised. Although this may introduce order-dependent bias, both measures were taken consecutively using standardised protocols; the test–retest reliability for manual height measurement was high indicating stable measurements. The study lacks a detailed analysis of how lighting conditions and clothing have affected the quality of depth maps. Direct sunlight and strong ambient light might affect the ToF sensor performance. This is one of the reasons for indoor scanning of children. ToF performance might differ between smartphone models with different camera and sensor qualities. The findings reported for CGM app's performance in this paper are with Huawei Pro P30 smartphone (phone used in CGM) with ToF sensor which consists of a Sony IMX516/316 image sensor and a Vertical-Cavity Surface-Emitting Laser (VCSEL) illumination unit, operating in the near-infrared (NIR) spectrum (typically 940 nm). This Sony image sensor cost approximately USD 35. Nevertheless, to overcome ToF sensor dependence, external sensors matching these specifications can be integrated to the phone with the CGM app to make it phone agnostic.

Despite these challenges, the study's strength lies in its application to real-world, field-based settings instead of strictly controlled clinical environments. This takes the study findings closer to field feasibility and effectiveness. Although data loss due to missed measurements was observed, the overall sample size remained adequate for inference. Moreover, the ability of the CGM app to generate automatic Z-scores based on WHO growth standards reduces the need for manual plotting and interpretation, addressing a common source of error in field anthropometry.^23^^,^^49^ Given its good accuracy and reliability, the CGM app holds promise for integration into nutrition monitoring programs in the future. Considering the latest technical requirements outlined in UNICEF's Target Product Profile (TPP 2025∗) for height measurement, the CGM app fulfils acceptable performance criteria, both for measurement accuracy and precision.

Its ability to automate data recording, minimise manual entry errors and provide real time analytics can improve decision making in malnutrition programs. However, further implementation studies are needed to optimise its performance, particularly in younger age groups and to ensure its robustness across diverse environmental and demographic contexts. The app might require further simplification and training to ensure seamless adoption by frontline health care workers in Nepal's healthcare system, where need for task shifting amid resource constraints are common.^32^ Cost effectiveness assessments considering minimal hardware dependencies and cloud-based solutions, could further support scalability in low resource settings.^24^^,^^27^^,^^47^

The CGM app demonstrated good accuracy in stunting detection and height measurement among children between 24 and 59 months of age in Nepal. The app shows promising results for use in field settings in the future. It is noteworthy that the 3D imaging technology could be a game changer for early detection of malnutrition among children in community setting. Currently, the CGM app could predict height and stunting. Future research and development must focus on estimation of body weight through 3D imaging which could help in detection of underweight, wasting and overweight/obesity in children, and thus, serve as a primary comprehensive malnutrition screening tool. With appropriate refinements and implementation strategies, the CGM app has the potential to transform anthropometric assessments, facilitating more efficient and standardised child growth monitoring in public health system. Further studies are required to evaluate the app among children 6–24 months of age which is a critically important age group within the first 1000 days framework for stunting prevention. In addition, future validation studies should assess CGM's performance across diverse population contexts and geographies to strengthen its generalisability and integration into routine growth monitoring systems.

## Contributors

This study was conceptualised by RM, SK, AG, MK, NA, and AM. Data collection and supervision were undertaken by SK, NA, MR, and SR with training and oversight by SK and project administration by MK and NA. Data curation was conducted by AG, AV, and NA. Formal analyses were performed by HS, DS, and AM, with methodological inputs from RM and AM. HS, DS, RM, and AM wrote the first draft of the manuscript. All authors contributed to review, revisions, and editing of the manuscript and approved the final version.

## Data sharing statement

The datasets generated and analysed during the current study are not publicly available due to confidentiality of the linked data. They are available from the corresponding author on reasonable request and following approval from competent ethics and administrative authorities.

## Declaration of interests

SK, AG, MK, NA, and SR were members of the CGM study team but were not involved in the formal data analysis for this manuscript. The authors declare no other competing interests.

## References

[1] Malnutrition. https://www.who.int/health-topics/malnutrition.

[2] Wells J.C.K., Marphatia A.A., Amable G. (2021). The future of human malnutrition: rebalancing agency for better nutritional health. Global Health.

[3] De Sanctis V., Soliman A., Alaaraj N., Ahmed S., Alyafei F., Hamed N. (2021). Early and long-term consequences of nutritional stunting: from childhood to adulthood. Acta Biomed.

[4] Ekholuenetale M., Barrow A., Ekholuenetale C.E., Tudeme G. (2020). Impact of stunting on early childhood cognitive development in Benin: evidence from Demographic and Health Survey. Egypt Pediatric Association Gaz.

[5] de Onis M., Branca F. (2016). Childhood stunting: a global perspective. Matern Child Nutr.

[6] Global nutrition report | country nutrition profiles - global nutrition report. https://globalnutritionreport.org/resources/nutrition-profiles/asia/southern-asia/nepal/.

[7] Levels and trends in child malnutrition: UNICEF/WHO/World Bank Group joint child malnutrition estimates: key findings of the 2023 edition. https://www.who.int/publications/i/item/9789240073791.

[8] Akseer N., Tasic H., Nnachebe Onah M. (2022). Economic costs of childhood stunting to the private sector in low- and middle-income countries. EClinicalMedicine.

[9] Galasso E., Wagstaff A. (2019). The aggregate income losses from childhood stunting and the returns to a nutrition intervention aimed at reducing stunting. Econ Hum Biol.

[10] Büttner N., Heemann M., De Neve J.W., Verguet S., Vollmer S., Harttgen K. (2023). Economic growth and childhood malnutrition in Low- and middle-income countries. JAMA Netw Open.

[11] Gupta P.M., Sivalogan K., Oliech R. (2023). Impact of anthropometry training and feasibility of 3D imaging on anthropometry data quality among children under five years in a postmortem setting. PLoS One.

[12] Taylor M., Tapkigen J., Ali I., Liu Q., Long Q., Nabwera H. (2023). The impact of growth monitoring and promotion on health indicators in children under five years of age in low- and middle-income countries. Cochrane Database Syst Rev.

[13] Mavinkurve M., Azriyanti A.Z., Jalaludin M.Y. (2021). The short child: importance of early detection and timely referrai. Malays Fam Physician.

[14] Lensoni L., Elmiyati E., Yulinar Y., Yahya M., Hanum U. (2023). Effectiveness of using the anthropometric stunting meter in children aged 24-59 months at the Lageun Health Center, ACEH Jaya District. JPPIPA.

[15] Huong L.T., Xuan L.T.T., Phuong L.H., Huyen D.T.T., Rocklöv J. (2014). Diet and nutritional status among children 24–59 months by seasons in a mountainous area of Northern Vietnam in 2012. Glob Health Action.

[16] Nosaka N., Anzai T., Wakabayashi K. (2024). Height status matters for risk of mortality in critically ill children. J Intensive Care.

[17] Hossain F.B., Shawon M.S.R., Al-Abid M.S.U., Mahmood S., Adhikary G., Bulbul M.M.I. (2020). Double burden of malnutrition in children aged 24 to 59 months by socioeconomic status in five South Asian countries: evidence from demographic and health surveys. BMJ Open.

[18] WHO child growth standards: training course on child growth assessment. https://www.who.int/publications/i/item/9789241595070.

[19] Fletcher R., Díaz X.S., Bajaj H., Ghosh-Jerath S. (2017). 2017 IEEE Global Humanitarian Technology Conference (GHTC).

[20] Corsi D.J., Perkins J.M., Subramanian S.V. (2017). Child anthropometry data quality from Demographic and Health Surveys, Multiple Indicator Cluster Surveys, and National Nutrition Surveys in the West Central Africa region: are we comparing apples and oranges?. Glob Health Action.

[21] WHO Multicentre Growth Reference Study Group (2006). Reliability of anthropometric measurements in the WHO Multicentre Growth Reference Study. Acta Paediatr Suppl.

[22] Conkle J., Ramakrishnan U., Flores-Ayala R., Suchdev P.S., Martorell R. (2017). Improving the quality of child anthropometry: manual anthropometry in the Body Imaging for Nutritional Assessment Study (BINA). PLoS One.

[23] Grellety E., Golden M.H. (2016). The effect of random error on diagnostic accuracy illustrated with the anthropometric diagnosis of malnutrition. PLoS One.

[24] Bougma K., Mei Z., Palmieri M. (2022). Accuracy of a handheld 3D imaging system for child anthropometric measurements in population-based household surveys and surveillance platforms: an effectiveness validation study in Guatemala, Kenya, and China. Am J Clin Nutr.

[25] Soller T., Huang S., Horiuchi S., Wilson A.N., Vogel J.P. (2023). Portable digital devices for paediatric height and length measurement: a scoping review and target product profile matching analysis. PLoS One.

[26] Kustiawan T.C., Nadhiroh S.R., Ramli R., Butryee C. (2022). Use of mobile app to monitoring growth outcome of children: a systematic literature review. Digit Health.

[27] Conkle J., Suchdev P.S., Alexander E., Flores-Ayala R., Ramakrishnan U., Martorell R. (2018). Accuracy and reliability of a low-cost, handheld 3D imaging system for child anthropometry. PLoS One.

[28] Conkle J., Keirsey K., Hughes A. (2019). A collaborative, mixed-methods evaluation of a low-cost, handheld 3D imaging system for child anthropometry. Matern Child Nutr.

[29] (2025). Wikipedia.

[30] Hansard M., Lee S., Choi O., Horaud R. (2013). https://link.springer.com/10.1007/978-1-4471-4658-2.

[31] Khandelwal Y., Arvind M., Kumar S. (2024). NurtureNet: a multi-task video-based approach for newborn anthropometry. arXiv.

[32] Chan D., Chua M.C., Hadimaja M., Mukherjee S., Wong J., Yap F. (2025). Artificial intelligence-driven anthropometric assessment for young children: evaluating the accuracy and practicality of a digital image-based length and weight prediction tool. BMJ Health Care Inform.

[33] Bilukha O., Couture A., McCain K., Leidman E. (2020). Comparison of anthropometric data quality in children aged 6-23 and 24-59 months: lessons from population-representative surveys from humanitarian settings. BMC Nutr.

[34] Rae S., Pullenayegum E., Ong F. (2025). Reliability of anthropometric measurement of young children with parent involvement. Child Obes.

[35] About: Rautahat district. https://dbpedia.org/page/Rautahat_District.

[36] UNICEF Supply Division Innovation Unit (2017). UNICEF target product profile Height/length measurement Device(s). UNICEF. https://www.unicef.org/supply/media/1291/file/Target%20product%20profile%20(TPP):%20Height%20length%20measurement%20device.pdf.

[37] United Nations Children’s Fund (2019). Evaluation of innovation in UNICEF work case Study: Height/Length measurement devices Project. UNICEF. https://www.unicef.org/evaluation/media/956/file/Height.

[38] WHO (2006). The WHO child growth standards. WHO. https://www.who.int/tools/child-growth-standards/standards.

[39] Namene J., Hunter C.J., Hodgson S. (2024). Reliability of anthropometric measurements of a digi-board in comparison to an analog height board in Namibian children under 5 years. Matern Child Nutr.

[40] Huang S., Conkle J., Homer C.S.E., Kounnavong S., Phongluxa K., Vogel J.P. (2023). Comparing the accuracy of an ultrasound height measurement device with a wooden measurement board among children aged 2–5 years in rural Lao People's Democratic Republic: a methods-comparison study. PLoS One.

[41] Recommendations for data collection, analysis and reporting on anthropometric indicators in children under 5 years old. https://www.who.int/publications/i/item/9789241515559.

[42] Addula S.R., Ali A. (2025). A novel permissioned blockchain approach for scalable and privacy-preserving IoT authentication. JCSRA.

[43] WHO Anthro Survey Analyser and other tools. https://www.who.int/tools/child-growth-standards/software.

[44] Cohen J.F., Korevaar D.A., Altman D.G. (2016). STARD 2015 guidelines for reporting diagnostic accuracy studies: explanation and elaboration. BMJ Open.

[45] Research agenda to address gaps in data collection, analysis and reporting on anthropometric indicators in children under 5 years old. https://www.who.int/groups/who-unicef-technical-expert-advisory-group-on-nutrition-monitoring/anthropometic-data-quality-briefs.

[46] Wagner D.R., Castañeda F., Bohman B., Sterr W. (2019). Comparison of a 2D iPad application and 3D body scanner to air displacement plethysmography for measurement of body fat percentage. J Hum Nutr Diet.

[47] Leidman E., Jatoi M.A., Bollemeijer I., Majer J., Doocy S. (2022). Accuracy of fully automated 3D imaging system for child anthropometry in a low-resource setting: effectiveness evaluation in malakal, South Sudan. JMIR Biomed Eng.

[48] Jefferds M.E.D., Mei Z., Palmieri M. (2022). Acceptability and experiences with the use of 3D scans to measure anthropometry of young children in surveys and surveillance systems from the perspective of field teams and caregivers. Curr Dev Nutr.

[49] Chanyarungrojn P.A., Lelijveld N., Crampin A. (2023). Tools for assessing child and adolescent stunting: lookup tables, growth charts and a novel appropriate-technology “MEIRU” wallchart - a diagnostic accuracy study. PLOS Glob Publ Health.

