# Supplementary material for: Accuracy of a smartphone-based 3D imaging tool for height measurement and stunting detection among children 24–59 months of age in Nepal: a validation study
Source: Lancet Reg Health Southeast Asia. 2026 Apr 24;48:100774. doi: 10.1016/j.lansea.2026.100774 (PMC13129374; doi:10.1016/j.lansea.2026.100774)
Supplement: Supplementary Material [file mmc1.pdf]

Supplementary File

Contents

Supplementary File 1. CGM Field Validation Protocol Version dated 05 September 2024 2
Supplementary File 2. Time-of-Flight (ToF) Sensor 7
Supplementary File 3. SMART Training 9
Supplementary File 4. Tables 12
Supplementary File 5. Figures 18

List of Tables

Table S4.1. Data collection plan as per days and health posts available in Nepal 12
Table S4.2. Profile of children who came to HP for their first measurement 12
Table S4.3. Profile of children who came to HP for repeat measurement by enumerator A 13
Table S4.4. Profile of children who came to HP for repeat measurement by enumerator B 13
Table S4.5. Availability of manual and CGM data 14
Table S4.6. Evaluation of accuracy and precision of CGM measurements. (n=75) 14
Table S4.7. Intra-rater reliability of CGM App and manual anthropometry for measurements of height (n=76) 15
Table S4.8. Inter-rater reliability of CGM App and manual anthropometry for measurements of height (n=75) 16
Table S4.9. Intercept, slope and residual standard deviation of Passing-Bablok regression, intercept of spline regression bias, lower and upper limit of agreement from the Bland-Altman plots (n=261) 16
Table S4.10. Agreement of CGM predicted height against gold standard (n=261) 17

List of Figures

Figure S5.1. Schematic for data collection for first and repeat measurement 18

Supplementary File 1. CGM Field Validation Protocol Version dated 05 September 2024

Diagnostic performance of Child Growth Monitoring (CGM) App, Nepal | Methods

Study Objective:

1. To evaluate the performance of CGM app for screening of stunting in children aged 24 to 59 months
2. To evaluate the performance of CGM app for precise measurement of height in children aged 24 to 59 months

Study Design: A validation study design for testing diagnostic performance of CGM

Study Site: The study will be conducted in Rautahat, a district in Nepal. Four Health Posts (HPs) have been identified for the study. Children aged 24 to 59 months within the catchment of these four health posts will be mobilized for the measurements.

Sample Size Calculation:

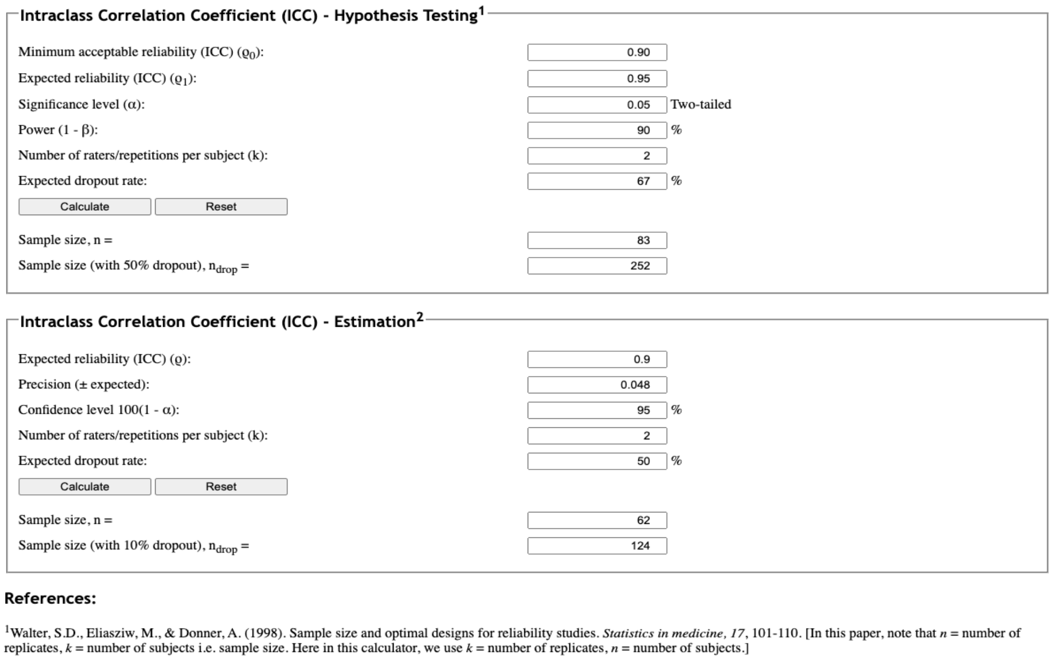

Intraclass Correlation Coefficient (ICC) - Hypothesis Testing[1]

Minimum acceptable reliability (ICC) (ϱ_0):	0.90	
Expected reliability (ICC) (ϱ_1):	0.95	
Significance level (α):	0.05	Two-tailed
Power (1 - β):	90	%
Number of raters/repetitions per subject (k):	2	
Expected dropout rate:	67	%
Calculate	Reset	
Sample size, n =	83	
Sample size (with 50% dropout), n_{drop} =	252	

Intraclass Correlation Coefficient (ICC) - Estimation[2]

Expected reliability (ICC) (ϱ):	0.9	
Precision (± expected):	0.048	
Confidence level 100(1 - α):	95	%
Number of raters/repetitions per subject (k):	2	
Expected dropout rate:	50	%
Calculate	Reset	
Sample size, n =	62	
Sample size (with 10% dropout), n_{drop} =	124	

References:

[1]Walter, S.D., Eliasziw, M., & Donner, A. (1998). Sample size and optimal designs for reliability studies. *Statistics in medicine, 17*, 101-110. [In this paper, note that *n* = number of replicates, *k* = number of subjects i.e. sample size. Here in this calculator, we use *k* = number of replicates, *n* = number of subjects.]

Figure S1.1. Sample size calculation for ICC Intra-rater and Inter rater

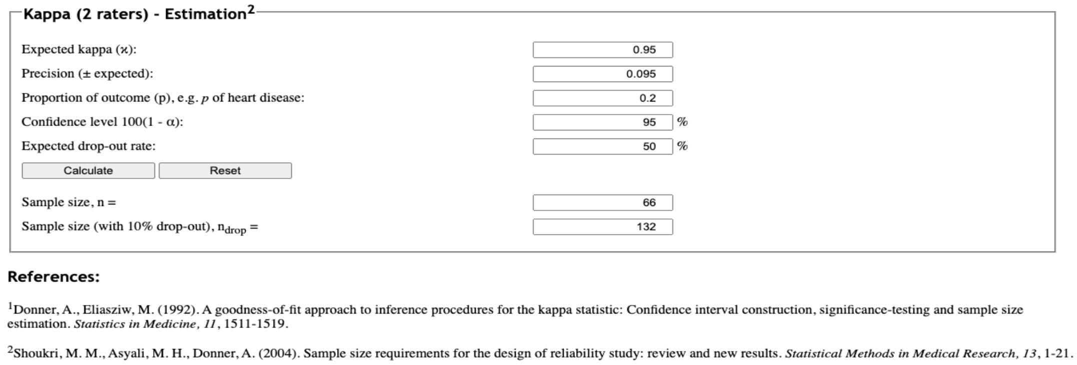

Kappa (2 raters) - Estimation[2]

Expected kappa (ϰ):	0.95	
Precision (± expected):	0.095	
Proportion of outcome (p), e.g. *p* of heart disease:	0.2	
Confidence level 100(1 - α):	95	%
Expected drop-out rate:	50	%
Calculate	Reset	
Sample size, n =	66	
Sample size (with 10% drop-out), n_{drop} =	132	

References:

[1]Donner, A., Eliasziw, M. (1992). A goodness-of-fit approach to inference procedures for the kappa statistic: Confidence interval construction, significance-testing and sample size estimation. *Statistics in Medicine, 11*, 1511-1519.

[2]Shoukri, M. M., Asyali, M. H., Donner, A. (2004). Sample size requirements for the design of reliability study: review and new results. *Statistical Methods in Medical Research, 13*, 1-21.

Figure S1.2. Sample size calculation for ICC for Kappa (Agreement between CGM measurements by two different enumerators)

For Diagnostic Performance (Sn, Sp, etc)

- Assuming 90% Sensitivity of CGM for detecting children with stunting, relative precision of 10% and 95% confidence level, and 20% data loss we would need 52 children with stunting. Assuming the prevalence of stunting to be about 20%, we would thus need to survey about 260 children. To round off, we have suggested a sample size of 300.

The planned sample size of 300 children aged 24 to 59 months looks adequate to:

- Compare agreement for detection of stunting using manual measurement by experts and CGM measurements in various combinations (inter/ intra). Please ensure that about 20% of the children measured at first instance and also during repeat measurement are stunted. That will take care of Kappa agreements
- At least 120 children (of which at least 25 should be stunted) of the 300 should go through repeat measurement, with at least 24 hours gap period from the time of their first measurement. This will enable us to confidently comment on the reliability of the app as well as the manual measurements.

Eligibility criteria for selection of children:

Inclusion criteria-

- The children should be aged 24-59-month-old. Preferably, this must be verified from some document of acceptable validity e.g. the birth certificate, immunization card, etc.
- All the children should be residents of the selected village
- The caregiver (parent/ guardian) should consent for the child's participation in the study both for first time as well as for repeat measurement.
- Children of any gender are eligible to participate in the study without over-representation of any particular gender (not more than 60%).

Exclusion criteria-

- The child is suffering from an illness and needs rest
- The child has a significant physical locomotor deformity
- The child is unwilling to cooperate and is difficult to manage or pacify

Discontinuation of participation

- The participation of the child can be discontinued if the caregiver wants to withdraw the consent for child' participation in the study at any point

How to choose kids and how many kids in a day?

- The villages with the largest population will be identified for enumeration
- An entire village will be considered as the catchment area
- The participant children will be selected through house-to-house visits
- If the requisite sample size is not met, then the adjacent villages will be identified for enumeration
- Efforts should be made to bring in children from all categories i.e., stunted, wasted, obese, healthy. Adequate number of stunted children should be brought for being measured in the Health Post (details of sample size have been provided above).

Method of Data Collection

- The data collection will be done over a period of 10 days i.e., 20-29th of October, 2024
- There will be 4 Health Posts (A, B, C and D). Each of the Health Post will have two trained enumerators/ experts of whom one would be taking the measurement and another one would be assisting.
-

- ***Activities during Day 1 to Day 5:***
    - During these first 5 Days, study activities will be functional at all the Health Posts and the team would take the anthropometric measurements of the children.
    - Each of the 4 Health Post (HP) will be measuring at least 15 children per day. These children will be the ones who would be measured for the first time.
    - Thus, each day a total of 60 children will be measured for the first time across the health posts and in 5 days' time, a total of 300 children will be measured. (15 children * 4 HP * 5 days). (Refer **Table S1.1)**
    - **Activity in each of the HP for every child during Day 1 to 5**: The child (say, Child A) will be first scanned using the CGM App by a trained expert (say, Expert A). Once the CGM scan is completed, Child A will then undergo manual measurement using the Gold Standard instruments by the same expert i.e., Expert A. The manual measurements will include measurements of height, weight and MUAC. Once the manual measurements are completed, the child leaves the HP. (Refer **Figure S1.3)**
- ***Activities during Day 6 to Day 9:***
    - During day 6 and 7, only two the Health Posts will be functional (say, HP A and C) and would take the repeat anthropometric measures of the children. While the other two HPs will remain closed (say, HP B and D), the Expert enumerators from these HPs will join the HPs A and C.
    - Similarly, during day 8 and 9, the other two the Health Posts will be functional (say, HP B and D) and would take the repeat anthropometric measures of the children. While the other two HPs will remain closed (say, HP A and C) and the Experts from these HPs will join the HPs B and D to take the measurements
    - Each of the Health Posts (HP) will be taking repeat measurements of at least 15 children per day from the group of children who were already measured during day 1 to 5. These children will be the ones who would be measured for the second time. At least 3 of these 15 children should be stunted.
    - Thus, each day a total of 30 children will be measured for the second time and in 4 days' time, a total of 120 children will be repeat measured. (15 children * 2 HP * 4 days). (Refer **Table S1.1)**
    - **Activity in each of the HP for every child during Day 6 to 9**: The child (say Child A) will come in the HP for repeat measurement. The Child A will first be scanned using the CGM App followed by manual measurement, by the trained expert who had done the measurements for the child the first-time s/he had visited the health post (i.e. Expert A). Then Child A will again be scanned using CGM by Expert B (i.e., the expert from a different Health Post that is closed during that day). Once the CGM scans are completed, Child A will undergo manual measurement using the physical instruments by the second expert i.e., Expert B. The manual measurements will include measurements of height, weight and MUAC. Once the manual measurements are completed, the child leaves the HP. (Refer **Figure S1.3)**

Table S1.1. Proposed data collection plan as per days available and Health Posts in Nepal

Health Post	Days for Data Collection									
	1	2	3	4	5	6	7	8	9	10
A	15F	15F	15F	15F	15F	15R	15R	××	××	Buffer Day to complete target sample size
B	15F	15F	15F	15F	15F	××	××	15R	15R	
C	15F	15F	15F	15F	15F	15R	15R	××	××	
D	15F	15F	15F	15F	15F	××	××	15R	15R	
Total	60F	60F	60F	60F	60F	30R	30R	30R	30R	
	300 F					120 R				

NOTE:
15F: At least 15 kids being measured for the first time in each of the Health Posts (F stands for the First measurement taken)
15R: At least 15 kids being measured for the second time in each of the Health Posts (R stands for Repeat measurement taken)
××: The particular Health Posts remains closed while the enumerators / experts join the functional Health Posts

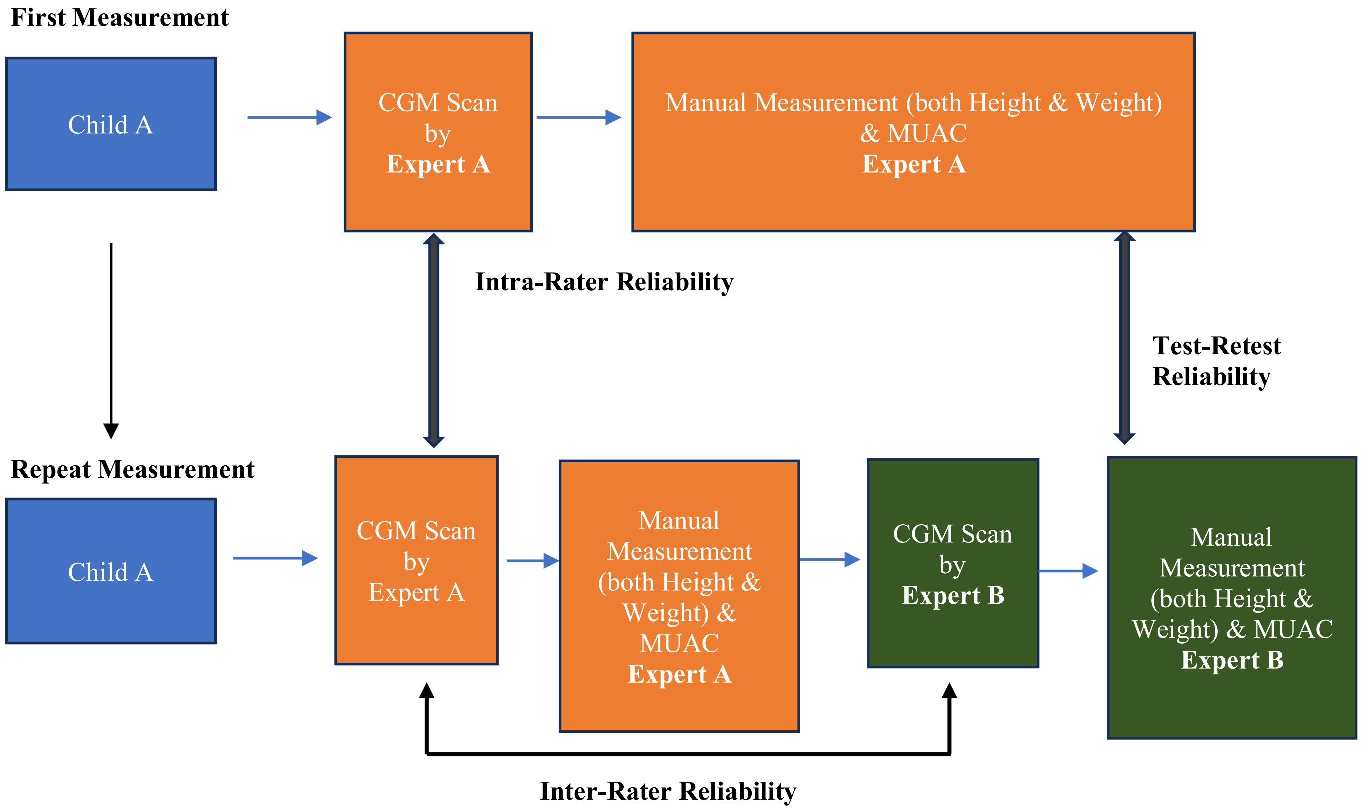

NOTE:

- All measurements must be noted/ recorded against the name of the Expert doing the measurement. This data required and is important for analysing inter and intra rater reliability
- Efforts should be made to bring in children from all categories. Stunted children should be purposively brought for being measured in the Health Post

Figure S1.3. Planned activity for Data collection in Nepal

Analysis Plan

- **Step 2 (Reliability of CGM)**
  - *Intra-rater reliability:* We will compute and comment on the scatter plots and intra-class correlation coefficient (ICC) for measurements taken by the SAME enumerator on the same child but on two different occasions.
  - *Inter-rater reliability:* We will compute and comment on the scatter plots and intra-class correlation coefficient (ICC) for measurements taken by DIFFERENT enumerators on the same child on the same occasion.
  - *Agreement:* Based on CGM measurements, we will compute and comment on the agreement between the two enumerators for detecting stunting by computing Cohen's Kappa.
  - *Variance assessment*: Evaluate variance between paired measurements using the Pitman test.
- **Step 3 (Validity of CGM)**
  - *Comparison of manual height measurement and CGM's predicted height (first measurement):* We will examine and comment on the Spearman-Brown Split-Half correlation coefficient, and the Intra-Class Correlation Coeffcient for Hypothesis testing.

- *Sensitivity, Specificity, Positive and Negative Predictive Values and Likelihood Ratios, Accuracy:* We will compute and comment on these by comparing the first CGM measurement with the manual gold standard measurements.
- *Precision and Accuracy of CGM measurements across enumerators:* Accuracy will be checked by doing a ROC curve analysis between the CGM measurements by DIFFERENT enumerators on the SAME child. We will also undertake Lin's Concordance Correlation Analysis for reviewing the precision and accuracy between the CGM measurements by different enumerators.

- **Step 4 (Assessment of Bias)**
  - Against manual gold standard, we will compute and comment on the bias, proportional bias and limits of agreement of CGM (first measurement) using Bland-Altman statistics, and on constant and proportional bias using Passing-Bablok Regression Analysis.
  - Comparison of profiles of children who underwent only manual measurement and those who underwent both manual and CGM first measurement, and those who underwent CGM single measurement versus repeat CGM measurements.
  - Systematic and proportional bias:
    - Evaluate bias using Cumulative Sum (CUSUM) testing.
    - Conduct spline regression to assess non-linearity and proportional bias.

Contingencies

- All above mentioned analysis planning has been done based on expectations of availability of data of sufficient quality and numbers.
- The plan for statistical analysis, choice of tests and commentary will vary based on data sufficiency, normality, level of interpretation, and satisfaction therewith.
- Analysis will be done using R Studio and Microsoft Excel spreadsheets.

Supplementary File 2. Time-of-Flight (ToF) Sensor

Time-of-Flight depth sensing and CGM scanning workflow

The Child Growth Monitor (CGM) application uses smartphones equipped with Time-of-Flight (ToF) depth sensors to capture three-dimensional information about the child during scanning. ToF sensors estimate distance by emitting near-infrared light signals and measuring the time taken for the reflected signal to return to the camera sensor. This process generates a depth map representing the distance between the device and different points on the child's body surface.

During scanning, the CGM application captures depth maps together with red-green-blue (RGB) images across four standardized poses. These images are processed using machine-learning algorithms that reconstruct a three-dimensional representation of the child's body. From this reconstruction, the application automatically estimates standing height and derives height-for-age z-scores based on World Health Organization growth standards.

The application operates on compatible smartphones with integrated depth sensors and is designed for use in field settings. Enumerators are trained in device positioning, child posture, and scan acquisition to ensure consistent image capture. Steps involved in scanning a child for stunting using CGM application have been summarised in Table S2.1.

Table S2.1. Steps involved in scanning a child for stunting using CGM application

Step	Description
Device setup	Smartphone mounted on tripod at fixed height
Child positioning	Child stands upright with feet together
Pose capture	Four standardized poses captured by CGM application
Image acquisition	Depth maps and RGB images recorded simultaneously
Processing	AI algorithm reconstructs 3D body model
Output	Estimated height and WHO-based HAZ classification

About TOF Sensor

The Time-of-Flight (ToF) sensor is a depth-sensing system that measures the distance by calculating the time it takes for the light to travel from the sensor to an object and return back.[1] ToF (Time-of-Flight) sensors can be categorized as Direct ToF (DToF) and Indirect ToF (IToF), based on how they measure the travel time of light. DToF uses short light pulses, whereas IToF employs continuous modulated waves. However, the ToF sensors built into smartphones typically use IToF, which measures the phase shift between the emitted and received modulated light. Usually, ToF sensors are integrated into smartphones to enhance photography quality and to support augmented reality (AR) applications. We are making use of the smartphone integrated ToF sensor for 3D scanning, i.e., capturing depth information along with RGB images.

Huawei Pro P30 smartphone's (phone used in CGM) ToF sensor consists of a Sony IMX516/316 image sensor and a Vertical-Cavity Surface-Emitting Laser (VCSEL) illumination unit, operating in the near-infrared (NIR) spectrum (typically 940 nm). The modulation frequency is multiple, typically ranging from 20 MHz to 100 MHz, and the operating range is 0.5 m to 4.0 m.

In the setup, the smartphone's ToF sensor emits a continuously modulated light signal toward a child standing against a plain wall. The distance from the smartphone placed on a tripod, with a constant angle between the

camera and the floor, to the child (approximately 1 m) and the wall (approximately 2 m) is kept constant within the operating range of the TOF sensor. Reflections from both the child's body and the wall are captured by the sensor. The smartphone then estimates the phase difference between the emitted and received signals to compute depth per pixel, i.e., the distance from the camera. Importantly, no extra calculation is required for depth map generation, as this is a built-in process of the smartphone's ToF sensor. Simultaneously with each depth map capture, a corresponding RGB image is automatically captured using the smartphone's camera.

No extra training is required for users when using a smartphone's built-in ToF sensor. Once the scan start button is clicked, ToF sensor automatically gets activated and starts capturing the depth. Training is only necessary to ensure the correct use of the CGM application, such as manual measurement entry, proper child positioning, and accurate placement of the smartphone during measurement. The CGM application provides feedback on the setting up distance, angle, and child positioning, making it user-friendly.

The functioning of the ToF sensor should be checked periodically to verify the correctness of the generated depth map. This is achieved by calibrating the camera, which is integrated into our application, and by viewing both the depth map and the corresponding RGB image on the server side. If a smartphone does not have a built-in ToF sensor, external integration would be required. Such integration was not part of our current workflow or application design.

Smartphones with integrated ToF (Time-of-Flight) sensors usually come in the mid-range to premium category. In India, their prices are ranging from about USD 268 to over USD 1073 depending on brand and features. Our current objective is to evaluate the usefulness of 3D scanning for measuring child height and applying it to stunting detection. Once a successful solution is established, it can be extended to low-range, budget-friendly devices either by externally integrating a ToF sensor or another depth-capturing sensor, or by employing software-based depth estimation.

CGM Height Estimation Workflow

Primarily, 3D scans (RGB images combined with depth maps) are captured and fed into Convolutional Neural Network (CNN) models to extract features and estimate height as a regression task. To ensure full body coverage, images of each child wearing minimal clothing are captured from multiple views—front, back, left, and right. These scans were collected specifically for height estimation, but the same approach could be extended to other perspectives such as classification of wasting, muac estimation etc... A sufficient dataset of children's measurements (manual height records along with 3D scans) is collected and divided into training and testing subsets.

The CNN model is trained on the training dataset (3D scans paired with manual measurements) using supervised learning, without exposing the test data. The trained model is then evaluated on the test dataset to assess its accuracy on unseen data. Once validated, this trained model is used for height estimation. Importantly, an additional dataset is collected independently for the purpose of validating the solution. This validation dataset is not used in either training or testing, ensuring an unbiased evaluation of the model's performance.

Reference

1. Hansard M, Lee S, Choi O, Horaud R. Time-of-Flight Cameras: Principles, Methods and Applications [Internet]. London: Springer; 2013 [cited 2026 Mar 19]. (SpringerBriefs in Computer Science). Available from: https://link.springer.com/10.1007/978-1-4471-4658-2 doi:10.1007/978-1-4471-4658-2

Supplementary File 3. SMART Training

The SMART training was an initial step in conducting the study. The training aimed to provide participants with the skills and knowledge required to gather data using the CGM app on phones and manual measurement techniques with height boards validated by UNICEF. The training followed the agenda detailed in the Table S3.1.

Table S3.1. Agenda for the three day SMART training of enumerators on anthropometric data collection using Child Growth Monitor (CGM) App and manual methods

S.No	Topic	Day of training
1	Overview of Anthropometry and The CGM App	1
2	Demonstration and Practice of Anthropometry on Children	1
3	Common Mistakes in Anthropometry, Calibration of Equipment, and CGM Phones	1
4	Practical Session on Anthropometry	1
5	Parent Counselling and Consent Collection	2
6	Verifying the Measurement Area	2
7	Preparing Children for Measurements	2
8	Setting up the Space for Manual and Scan Measurements	2
9	Marking Distances for Scan Setup	2
10	Tripod and Phone Setup	2
11	Child Positioning and Child Scanning (Step-by-Step Walkthrough of App)	2
12	Scanning Guidelines and Checklist	2
13	Dummy Data Entry Practice and CGM Scans	2
14	Dummy Standardization Test with Manual Measurements and CGM Scans on Real Children	2
15	Real Standardization Test	3

Profile of Trainers

The training was conducted by two experienced trainers:

1. **Lead trainer:** The head of the project, holding a Master's in Public Health and a certified survey manager with eight years of experience conducting surveys and six years of global experience in conducting standardization tests. This trainer provided training on data collection and survey execution.
2. **Technical trainer:** A professional designer, holding a Master's in Visual Design and Communication with over 15 years of experience in design. This trainer provide training on phone handling, child scan capture, and ensuring high-quality scans, including appropriate phone setup, background management, child positioning, and data uploading processes.

Recruitment Process of Participants for Training

- **Job advertisement:** The role for data collector was advertised in local newspapers and web portals.
- **Selection criteria for interview:** Minimum higher education in nutrition, public health, or social sciences. Fluency in local languages (e.g., Nepali) was essential. Experience in conducting anthropometric or household-level data collection was desirable. Candidates with strong

communication, interpersonal skills, and technical knowledge of measurement techniques were prioritized.

- **Shortlisting:** Applications were reviewed based on qualifications and experience, with preference given to those involved in past health or nutrition programs.
- **Assessment and interviews:** Candidates were evaluated through interviews for interpersonal and language skills. Practical tests were conducted to assess their understanding of survey methodologies.
- **Selection criteria for training participation:** Candidates were assessed on technical competence, cultural sensitivity, reliability, and teamwork skills to ensure they could effectively collaborate with others.

Training Process

Once selected, participants underwent comprehensive training, which included:

- **Technical training:** Detailed orientation on SMART methodology and anthropometric measurement techniques, with hands-on practice using real equipment.
- **Practice exercise:** A dedicated practice session focused on taking gold-standard manual measurements, calibrating equipment, and setting up phones for the scanning process.
- **Field testing:** Pilot surveys were conducted to assess participant readiness and address skill gaps.
- **Standardization tests:** Participants underwent accuracy tests for anthropometric measurements to ensure they met predefined standards.
- **Evaluation:** Selection of enumerators was based on accuracy tests (Technical Error Measurement (TEM)). Out of 10 participants trained, 8 successfully passed the TEM assessment. Two participants were disqualified with TEM scores of 0.98 and 0.85, exceeding the acceptable TEM cut-off for height measurements (>0.6).

Standardization of Training

Following the completion of training, the study was conducted during which test-retest reliability of manual measurements was assessed. Results showed:

- **Test-retest reliability:** Measurements taken by two different enumerators on the same child resulted in an ICC of 0.949 (95% CI: 0.928–0.964; $p < 0.001$; n=122) and paired t-test showed no significant value (p=0.597).
- **Scatter plot analysis:** The scatter plot displayed strong alignment in height measurements, with minimal outliers, supporting the reliability of manual measurements. (Figure S3.1)

Based on these results, the training process was confirmed to be standardized and effective.

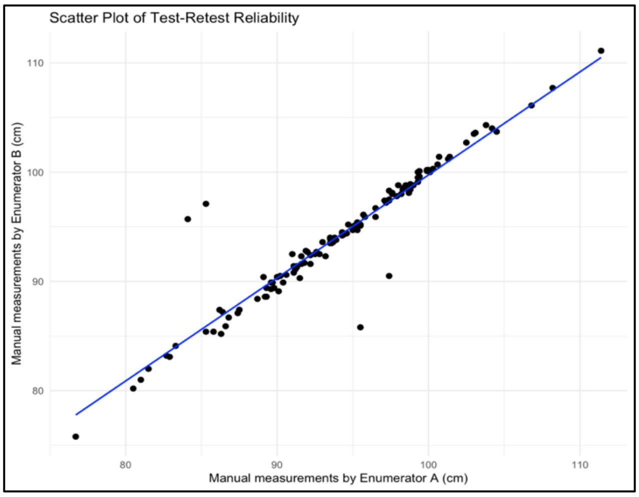

Figure S3.1. Scatter plot of manual height measurements between two enumerators

Supplementary File 4. Tables

Table S4.1. Data collection plan as per days and health posts available in Nepal

Health Post	Days for Data Collection									
	**1**	**2**	**3**	**4**	**5**	**6**	**7**	**8**	**9**	**10**
**I**	15F	15F	15F	15F	15F	15R	15R	××	××	Buffer Day to complete target sample size
**II**	15F	15F	15F	15F	15F	××	××	15R	15R	
**III**	15F	15F	15F	15F	15F	15R	15R	××	××	
**IV**	15F	15F	15F	15F	15F	××	××	15R	15R	
**Total**	60F	60F	60F	60F	60F	30R	30R	30R	30R	
	**300 F**					**120 R**				

NOTE:
15F: At least 15 kids being measured for the first time in each of the Health Posts (F stands for the First measurement taken)
15R: At least 15 kids being measured for the second time in each of the Health Posts (R stands for Repeat measurement taken)
××**:** The particular Health Posts remains closed while the enumerators / experts join the functional Health Posts

Table S4.2. Profile of children who came to HP for their first measurement

Profile	CGM data		Manual data		CGM and manual both data	Either CGM or manual data missing
	**Available data n (%)**	**No data n (%)**	**Available data n (%)**	**No data n (%)**	**Available data n (%)**	**No data n (%)**
**Gender**						
Girls	134 (51·0)	24 (51·1)	158 (51·1)	··	132 (50·6)	25 (51·0)
Boys	129 (49·0)	23 (48·9)	151 (48·9)	01 (100)	129 (49·4)	24 (49·0)
**Age (in months)**						
24-35	43 (16·3)	17 (36·2)	60 (19·4)	··	41 (15·7)	18 (36·7)
36-47	123 (46·8)	13 (27·7)	135 (43·7)	··	123 (47·1)	13 (26.5)
48-59	97 (36·9)	17 (36·2)	114 (36·9)	01 (100)	97 (37·2)	18 (36.7)
**Stunting categories**						
Not Stunted	179 (68·1)	47 (100)	198 (64·1)	01 (100)	167 (64.0)*	49 (100)
Moderately Stunted	72 (27·4)		91 (29·4)		80 (30.7)*	
Severely Stunted	12 (4·6)		20 (6·5)		14 (5.4)*	
**Total**	**263 (100)**	**47 (100)**	**309 (100)**	**01 (100)**	**261 (100)**	**49 (100)**

*Stunting categorization based on manual measurements

Table S4.3. Profile of children who came to HP for repeat measurement by enumerator A

Profile	CGM data		Manual data		CGM and manual both data	Either CGM or manual data missing
	Available data n (%)	No data n (%)	Available data n (%)	No data n (%)	Available data n (%)	No data n (%)
**Gender**						
Girls	45 (47·9)	10 (35·7)	55 (45·1)	··	45 (47·9)	10 (35·7)
Boys	49 (52·1)	18 (64·3)	67 (54·9)	··	49 (52·1)	18 (64·3)
**Age (in months)**						
24-35	17 (18·1)	8 (28·6)	25 (20·5)	··	17 (18·1)	8 (28·6)
36-47	41 (43·6)	13 (46·4)	54 (44·3)	··	41 (43·6)	13 (46·4)
48-59	36 (38·3)	07 (25·0)	43 (35·2)	··	36 (38·3)	07 (25·0)
**Stunting categories**						
Not Stunted	57 (60·6)	28 (100)	71 (58·2)	··	52 (55.3)*	28 (100)
Moderately Stunted	29 (30·9)		40 (32·8)	··	34 (36.2)*	
Severely Stunted	08 (8·5)		11 (9·0)	··	08 (8.5)*	
**Total**	**94 (100)**	**28 (100)**	**122 (100)**	··	**94 (100)**	**28 (100)**

*Stunting categorization based on manual measurements

Table S4.4. Profile of children who came to HP for repeat measurement by enumerator B

Profile	CGM data		Manual data		CGM and manual both data	Either CGM or manual data missing
	Available data n (%)	No data n (%)	Available data n (%)	No data n (%)	Available data n (%)	No data n (%)
**Gender**						
Girls	42 (41·6)	08 (36·4)	56 (45·5)	··	42 (41·6)	08 (36·4)
Boys	59 (58·4)	14 (63·6)	67 (54·5)	··	59 (58·4)	14 (63·6)
**Age (in months)**						
24-35	18 (17·8)	07 (31·8)	25 (20·3)	··	18 (17·8)	07 (31·8)
36-47	46 (45·5)	08 (36·4)	54 (43·9)	··	46 (45·5)	08 (36·4)
48-59	37 (3·6)	07 (31·8)	44 (35·8)	··	37 (36·6)	07 (31·8)
**Stunting categories**						
Not Stunted	64 (63·4)	22 (100)	72 (58·5)	··	62 (61.4)*	22 (100)
Moderately Stunted	30 (29·7)		37 (30·1)	··	28 (27.7)*	
Severely Stunted	07 (6·9)		14 (11·4)	··	11 (10.9)*	
**Total**	**101 (100)**	**22 (100)**	**123 (100)**	··	**101 (100)**	**22 (100)**

*Stunting categorization based on manual measurements

Table S4.5. Availability of manual and CGM data

Day	Enumerator	Not available n (%)	Available n (%)	Total
**Manual data**				
First	A	21 (6·4)	309 (93·6)	330 (100)
Repeat	A	3 (2·4)	122 (97·6)	125 (100)
	B	2 (1·6)	123 (98·4)	125 (100)
**CGM data**				
First	A	67 (20·3)	263 (79·7)	330 (100)
Repeat	A	31 (24·8)	94 (75·2)	125 (100)
	B	24 (19·2)	101 (80·8)	125 (100)

Table S4.6. Evaluation of accuracy and precision of CGM measurements. (n=75)

Parameter	Enumerator A	Enumerator B
**Receiver Operating Characteristic (ROC) analysis**		
**Area under the curve (AUC)**	0·881 (95% CI: 0·808- 0·955)	0·848 (95% CI: 0·760-0·937)
**Maximum Youden's index**	64	59·5
**Optimal threshold**	95·5	95·8

Table S4.7. Intra-rater reliability of CGM App and manual anthropometry for measurements of height (n=76)

Profile	Mean height (cm)		Technical Error of Measurement (TEM) (cm)		Relative TEM (%TEM)		Coefficient of reliability in technical error of measurement (R)		Intraclass Correlation Coefficient (ICC) (95%CI)		Paired t test (p-value)	
	**CGM**	**Manual**	**CGM**	**Manual**	**CGM**	**Manual**	**CGM**	**Manual**	**CGM**	**Manual**	**CGM**	**Manual**
**Overall**	94·3	93·9	0·5	0·3	0·6	0·3	0·990	0·997	0·990 (0·984-0·994)*	0·997 (0·996-0·998)*	0·837	0·609
**Gender**												
Boys (n=40)	95·2	94·7	0·5	0·3	0·5	0·3	0·993	0·998	0·993 (0·988-0·996)*	0·998 (0·996-0·999)*	0·787	0·090
Girls (n=36)	93·5	93·1	0·6	0·3	0·6	0·3	0·983	0·996	0·983 (0·967-0·991)*	0·998 (0·996-0·999)*	0·610	0·335
**Age (in months)**												
24-35 months (n=12)	88·6	88·1	0·5	0·3	0·6	0·3	0·990	0·998	0·991 (0·969-0·997)*	0·998 (0·992-0·999)*	0·409	0·084
36-47 months (n=35)	93·9	93·4	0·5	0·2	0·6	0·3	0·986	0·997	0·986 (0·972-0·993)*	0·997 (0·994-0·998)*	0·709	0·440
48-59 months (n=29)	97·3	97	0·5	0·3	0·5	0·3	0·983	0·994	0·983 (0·965-0·992)*	0·994 (0·987-0·997)*	0·636	0·693

* statistically significant p<0.05

Table S4.8. Inter-rater reliability of CGM App and manual anthropometry for measurements of height (n=75)

Profile	Mean height (cm)		Technical Error of Measurement (TEM) (cm)		Relative TEM (%TEM)		Coefficient of reliability in technical error of measurement (R)		Intraclass Correlation Coefficient (ICC) (95%CI)		Paired t test (p-value)	
	CGM	Manual	CGM	Manual	CGM	Manual	CGM	Manual	CGM	Manual	CGM	Manual
**Overall**	94·7	94·3	0·5	0·7	0·5	0·7	0·992	0·985	0·992 (0·987-0·995)*	0·985 (0·977-0·991)*	0·329	0·156
**Gender**												
Boys (n=42)	96	95·8	0·5	0·2	0·5	0·3	0·992	0·998	0·992 (0·985-0·996)*	0·998 (0·997-0·999)*	0·008*	0·599
Girls (n=33)	93·1	92·4	0·5	1	0·5	1·1	0·991	0·959	0·991 (0·982-0·996)*	0·959 (0·919-0·979)*	0·099	0·188
**Age (in months)**												
24-35 months (n=13)	89·4	88·8	0·5	0·3	0·6	0·3	0·991	0·998	0·992 (0·974-0·997)*	0.998 (0·993-0·999)*	0·117	0·491
36-47 months (n=33)	94·3	93·8	0·4	0·3	0·4	0·3	0·992	0·996	0·992 (0·985-0·996)*	0·996 (0·992-0·998)*	0·337	0·439
48-59 months (n=29)	97·6	97·2	0·6	1·1	0·6	1·1	0·985	0·951	0·985 (0·969-0·993)*	0·952 (0·901-0·977)*	0·727	0·963

* statistically significant p<0.05

Table S4.9. Intercept, slope and residual standard deviation of Passing-Bablok regression, intercept of spline regression bias, lower and upper limit of agreement from the Bland-Altman plots (n=261)

Methods compared	Passing-Bablok regression#			Spline regression		Bland-Altman plot			Pitman's Test	
	Intercept (95% CI)	Slope (95% CI)	RSD mean% (SD %)	Intercept (95% CI)	p-value	Bias (95% CI)	LoA (95% CI)	UoA (95% CI)	r (95% CI)	p-value
CGM/ Manual	3·7 (2 to 5·3)*	0·96 (0·95 to 0·98)	1 (16·6)	77·9 (76·9 to 78·8)*	< 0·001	0·3 (0·2 to 0·4)*	-1·6 (-1·8 to -1·4)	2·2 (2 to 2·4)	-0·325 (-0·429 to -0·212)	< 0·001

*statistically significant p<0.05
#Passing-Bablok regression was applied but CUSUM test revealed a significant deviation from linearity (p<0·001)
RSD: Residual standard deviation; CI: Confidence Interval; LOA: Lower limit of agreement; UOA: Upper limit of agreement.

Table S4.10. Agreement of CGM predicted height against gold standard (n=261)

Intra-Class Correlation Coefficient (ICC)			Spearman-Brown Split-Half coefficient	
**ICC**	**95% CI**	**p-value**	**Coefficient**	**95% CI**
0·986	0·982-0·989	< 0·001	0·994	0·990-0·995

Supplementary File 5. Figures

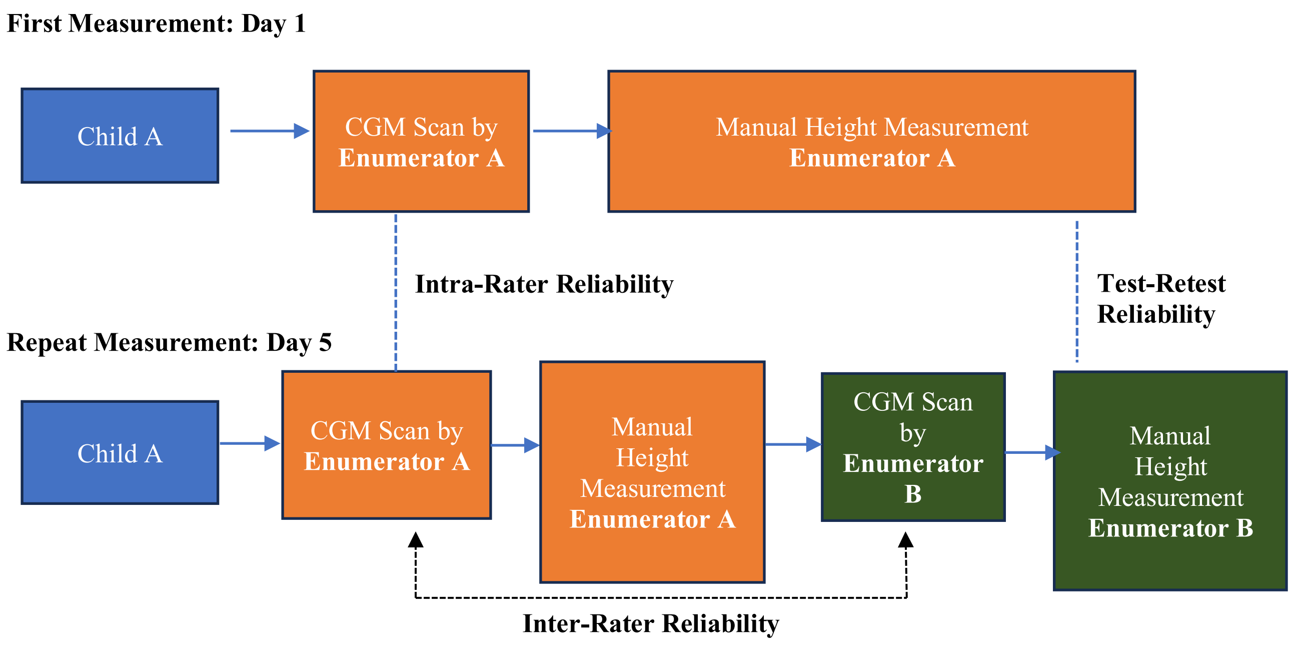

Figure S5.1. Schematic for data collection for first and repeat measurement
